# Editorial: Modulation of Stomatal Response by Elevated CO_2_ in Plants Under Drought and Heat Stress

**DOI:** 10.3389/fpls.2022.843999

**Published:** 2022-02-07

**Authors:** Xiangnan Li, Jairo A. Palta, Fulai Liu

**Affiliations:** ^1^Key Laboratory of Mollisols Agroecology, Northeast Institute of Geography and Agroecology, Chinese Academy of Sciences, Changchun, China; ^2^The UWA Institute of Agriculture, and School of Agriculture and Environment, The University of Western Australia, Perth, WA, Australia; ^3^CSIRO Agriculture and Food, Wembley, WA, Australia; ^4^Department of Plant and Environmental Sciences, Faculty of Science, University of Copenhagen, Tåstrup, Denmark

**Keywords:** climate change, CO_2_ elevation, drought, heat, hydraulic integrity, stomatal conductance, water-use efficiency

Understanding the physiological mechanisms regulating plant water relations is essential for sustainable crop production in future warmer, drier and elevated CO_2_ climate. Stomatal morphology and behavior plays a critical role in controlling plant water use though the interaction of elevated CO_2_ (*e*[CO_2_]), drought and heat stress. The Research Topic “*Modulation of Stomatal Response by Elevated CO*_2_ *in Plants Under Drought and Heat Stress*” comprises five original Research Topics and focuses on understanding the mechanisms regulating leaf gas-exchange and water-use efficiency across different crop species as influenced by drought and/or heat stress under *e*[CO_2_].

In a tropical forage legume, Habermann et al. examined the performance of stomatal properties of *S. capitata* and their consequence to plant function under *e*[CO_2_] and high temperature. Using the Trop-T-FACE facility that combines a FACE (free-air carbon dioxide enrichment) and a T-FACE (free-air temperature-controlled enhancement) system, the effects of *e*[CO_2_], heat stress and their combination on stomatal properties, leaf gas-exchange, starch content, PSII photochemistry, and water-use efficiency were evaluated. It was found that the effect of *e*[CO_2_] on stomatal aperture did not change by a warmer environment, while the combination of *e*[CO_2_] × heat stress significantly improved the growth performance, indicating that *S. capitata* is an important species in maintaining grassland productivity under predicted climate change scenario. Horticultural species grown in greenhouse may have different response to *e*[CO_2_] and drought stress. Li et al. conducted a greenhouse experiment with two tomato genotypes, the *Ailsa Craig* and its ABA-deficient mutant, *flacca*, to determine the responses of plant hydraulic integrity under *e*[CO_2_] and drought stress. It was found that *e*[CO_2_] could downregulate the expression of genes encoding plasma membrane intrinsic proteins (PIPs) in leaves and roots, which coincided with the lowered leaf and root hydraulic conductivity. This effect was ABA-dependent. Likewise, severe drought stress could also downregulate *PIPs* in leaves, which correlated with the dramatic decrease in leaf hydraulic conductivity. However, the regulation of *PIPs* by drought stress varied in roots as most of *PIPs* were downregulated; whereas *PIP2;1* was upregulated. These effects of drought stress were ABA-independent. When drought stress became severe, its effect on plant hydraulic conductivity could override the effects of *e*[CO_2_], indicating that *e*[CO_2_] might disturb ABA-mediated drought responses. Another original research paper by Torralbo et al. highlighted the roles of aquaporins in crop plant hydraulic responses to *e*[CO_2_] as they evaluated the relationships between leaf gas-exchange rate and the expression of genes involving CO_2_ and H_2_O diffusion in durum wheat (*Triticum durum* L.) grown under different types of nitrogen fertilizer at two atmospheric CO_2_ concentrations. Under ambient CO_2_ (*a*[CO_2_]), ammonium nutrition led to toxic effects with stress symptoms and reductions in stomatal conductance and leaf photosynthesis, affecting plant growth. However, under *e*[CO_2_], the reduction in leaf photosynthesis disappeared and stomatal conductance together with the expression of water, ammonium, and CO_2_ transporters (*TIP1, AMT2.1* and *PIP1.1*, respectively) were maintained at levels similar to the control plants. This indicates that ammonium- and ammonium nitrate supply were able to increase photosynthetic rate, which was associated to a high leaf protein content in the absence of a stress triggered by *e*[CO_2_].

Many studies have focused on the effects of *e*[CO_2_] on plant growth and physiology within a single generation, yet the long-term effect of *e*[CO_2_] over multiple generations, which will be the case for plants grown in future climate, has received rare attention. In a long-term study using a free-air *e*[CO_2_] system, Holohan et al. evaluated the differential capacity among several plant species to acclimate their intrinsic water-use efficiencies (WUE_i_) in response to multigenerational exposure to *e*[CO_2_]. It was found that long-term exposure to *e*[CO_2_] influenced the dynamic control of WUE_i_ in the first filial generations of all species, as well as an unequal ability to adapt to changes in the CO_2_ growth environment. However, the ability to increase WUE_i_ does not necessarily translate to an ecological advantage in diverse species mixtures. In another study with various plant species, Zhang et al. proposed a geometric constraint in analyzing the relationship between stomatal density (SD) and size (SS) or length based on machine learning algorithm, including data collection, slope comparison, comparison of the geometric constraint with non-geometric effect and variation partitioning and partial differential equation. Their results demonstrated that the higher geometric constraint likely caused the SD-SS relationship to be inevitably non-linear and negative. Meanwhile, they highlighted that a lower geometric constraint seems to extend the upper range of SD in angiosperm species and hence enable them to exploit a wide range of environments as compared to the pteridophyta and gymnosperms.

Figure 1 presented the roles of stomata and its relationship with other physiological processes in response to the interaction of *e*[CO_2_] × drought × heat stress. There is still a gap in exploring the bio-physiological mechanisms regulating stomatal aperture and whole-plant hydraulic integrity when grown under future drier, warmer and elevated CO_2_ climates. We hope this Research Topic encourage researchers to go further in this research area, contributing to an enhanced resilience of crops to climate change, and escorting a sustainable crop production and global food security.

**Figure 1:**
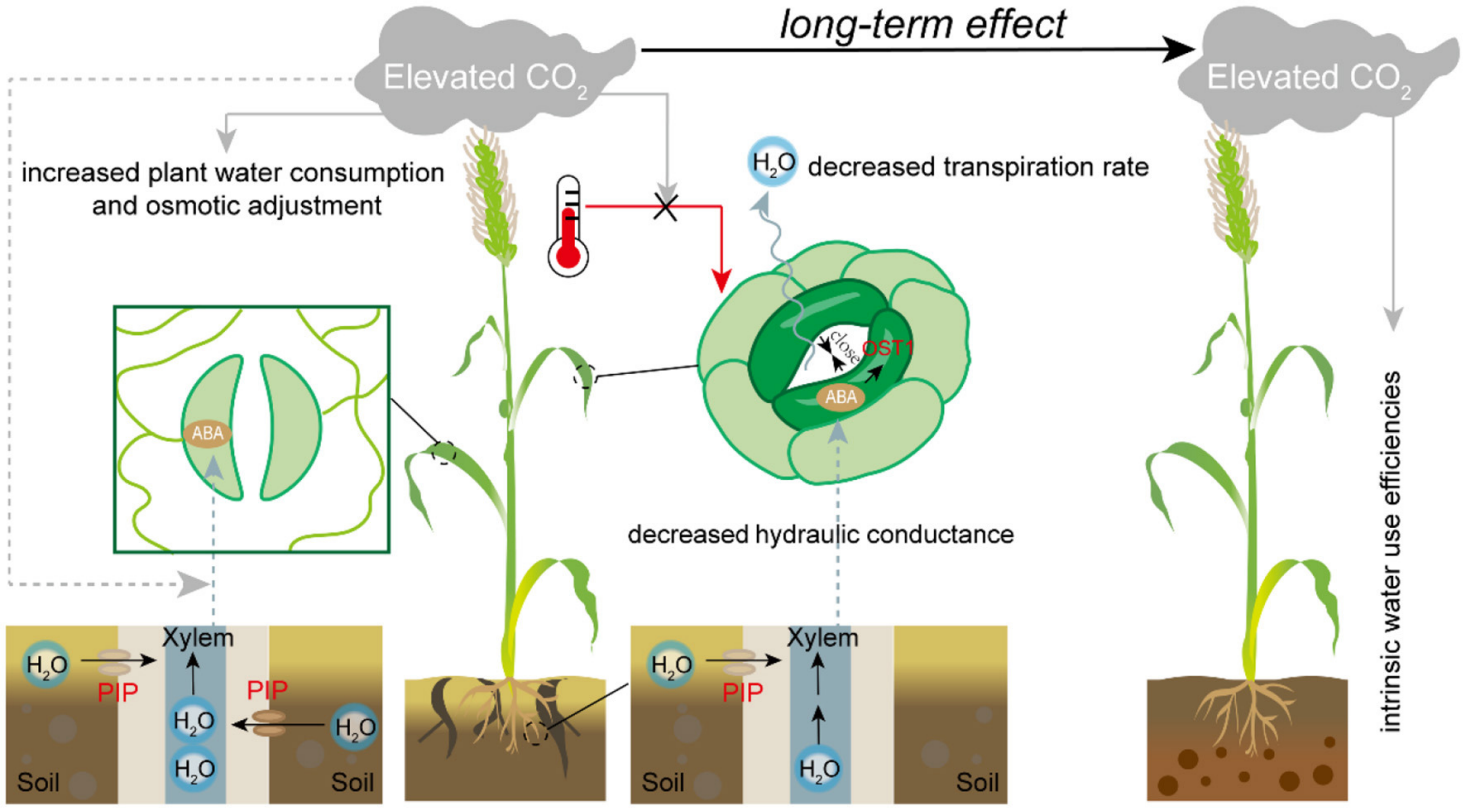
The roles of stomata and its relationships with other physiological processes in the responses to interactions of *e*[CO_2_], drought, and/or heat stress. The figure shows the modulation of stomatal response by *e*[CO_2_] in plants under drought and heat stress. Under a warmer condition, *e*[CO_2_] has no effect on stomatal opening; however, under drought stress the leaf and root hydraulic conductance, as well as transpiration rate, are decreased, which is coupling with the modulated transcriptional levels of *PIPs* and *OST1*. However, some *PIPs* are not influenced by *e*[CO_2_], thereby triggering the possibility that *e*[CO_2_] disturbs ABA-mediated drought responses. In addition, the long-term *e*[CO_2_] significantly influence the WUE_i_ in the first filial generation.

## Author Contributions

All authors listed have made a substantial, direct, and intellectual contribution to the work and approved it for publication.

## Funding

This research was funded by the Strategic Priority Research Program of the Chinese Academy of Sciences (XDA28020400), National Natural Science Fund for Excellent Young Scholars (31922064), CAS Pioneer Hundred Talents Program (C08Y194), and the Science and Technology Development Program of Jilin Province (20190201118JC and 20210402036GH).

## Conflict of Interest

The authors declare that the research was conducted in the absence of any commercial or financial relationships that could be construed as a potential conflict of interest.

## Publisher's Note

All claims expressed in this article are solely those of the authors and do not necessarily represent those of their affiliated organizations, or those of the publisher, the editors and the reviewers. Any product that may be evaluated in this article, or claim that may be made by its manufacturer, is not guaranteed or endorsed by the publisher.

